# Presence of scar by late gadolinium enhancement is a strong predictor of events in Chagas Heart Disease

**DOI:** 10.1186/1532-429X-16-S1-P343

**Published:** 2014-01-16

**Authors:** Gustavo J Volpe, Henrique T Moreira, Henrique S Trad, Katherine Wu, Maria Fernanda Braggion-Santos, Marcel Koenigkam Santos, Benedito C Maciel, Antonio T Pazin, José A Marin-Neto, Joao A Lima, Andre Schmidt

**Affiliations:** 1 Clinical Medicine/Division of Cardiology, HCFMRP-USP, Ribeirao Preto, São Paulo, Brazil; 2 Cardiology, Johns Hopkins Hospital, Baltimore, Maryland, USA; 3 Clinical Medicine/Radiology, HCFMRP-USP, Ribeirao Preto, Sao Paulo, Brazil; 4 Clinical Medicine/Emergency, HCFMRP-USP, Ribeirao Preto, Sao Paulo, Brazil

## Background

Chagas Heart Disease (CHD) is related to myocardial fibrosis (MF) and has a well-known relationship with sudden cardiac death (SCD). Cardiac magnetic resonance (CMR) can assess MF by late gadolinium enhancement (LGE) sequences. This study seeks to evaluate the presence of scar by LGE as a predictor of adverse outcomes in a CHD cohort.

## Methods

A total of 121 patients with CHD disease (52.1% female; 54.5 ± 13 years-old) from Ribeirao Preto Clinical Hospital (Sao Paulo, Brazil) were included. A CMR was performed at the enrollment from October/2009 to March/2013 in a single 1.5T scanner (Achieve, Phillips, the Netherlands), including SSFP cines at the vertical and horizontal long axis, and a stack of the short axis. LGE sequences were performed after 10.1 ± 1.5 min gadolinium contrast (0.2 mmol/kg) in the same cine positions, with positive LGE visually assessed and quantified for core and grey zone. All analysis was performed with Mass Research version software (Leiden University, the Netherlands). Any death, pacemaker (PC) implant and heart failure hospitalization (HFH) was considered an event during the follow-up. Kaplan-Meier curves, log-rank analysis and Cox regression models were utilized at the statistical analysis.

## Results

**Figure 1:**
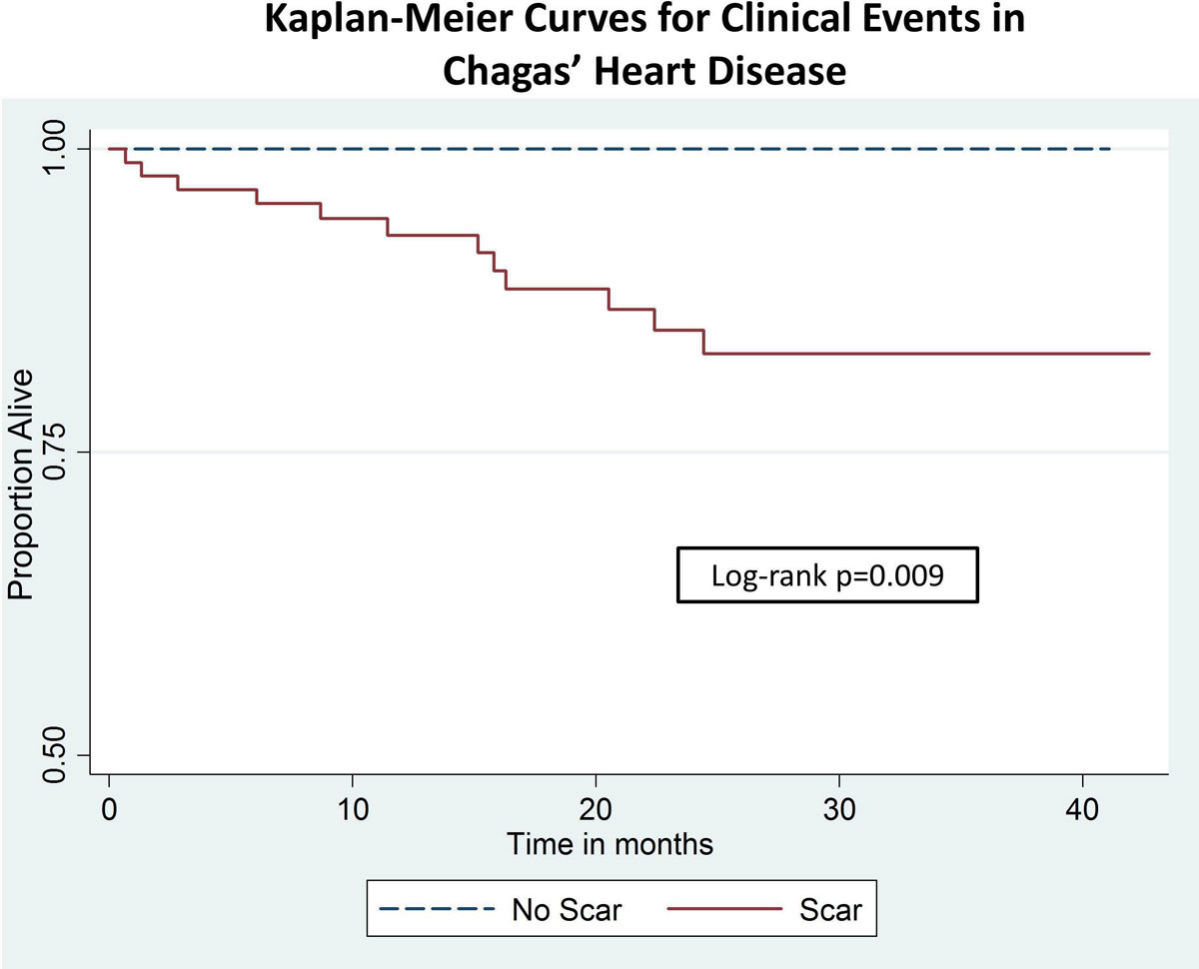


There was scar in 78.5% of the patients, with the lateral, inferolateral and inferior walls as the most common areas of fibrosis. The ones with positive LGE had lower left ventricular (LV) ejection fraction, and higher LV end diastolic volume and LV mass. No difference in other cardiovascular risk factors was noted. After 24.5 ± 13 months of follow-up, 12 events occurred (4 deaths, 4 PC and 4 HFH). No events were observed among patients without MF. At the survival analysis, patients with scar had a lower event-free time when compared to the ones without scar (Figure 1). After adjusting for age, gender, hypertension, diabetes, and LV ejection fraction, presence of more than 5% of core scar (HR 0.2 per %, p = 0.017) and more than 5% of grey-zones (HR 2.19 per %; p < 0.001) were independent predictors of events.

## Conclusions

In patients with CHD, presence of scar by LGE is a strong predictor of adverse events. The amount of core scar and grey zones were also independent predictors of events.

## Funding

The Study was partially funded by Coordenacao de Aperfeicoamento Pessoal (CAPES).

**Figure 2:**
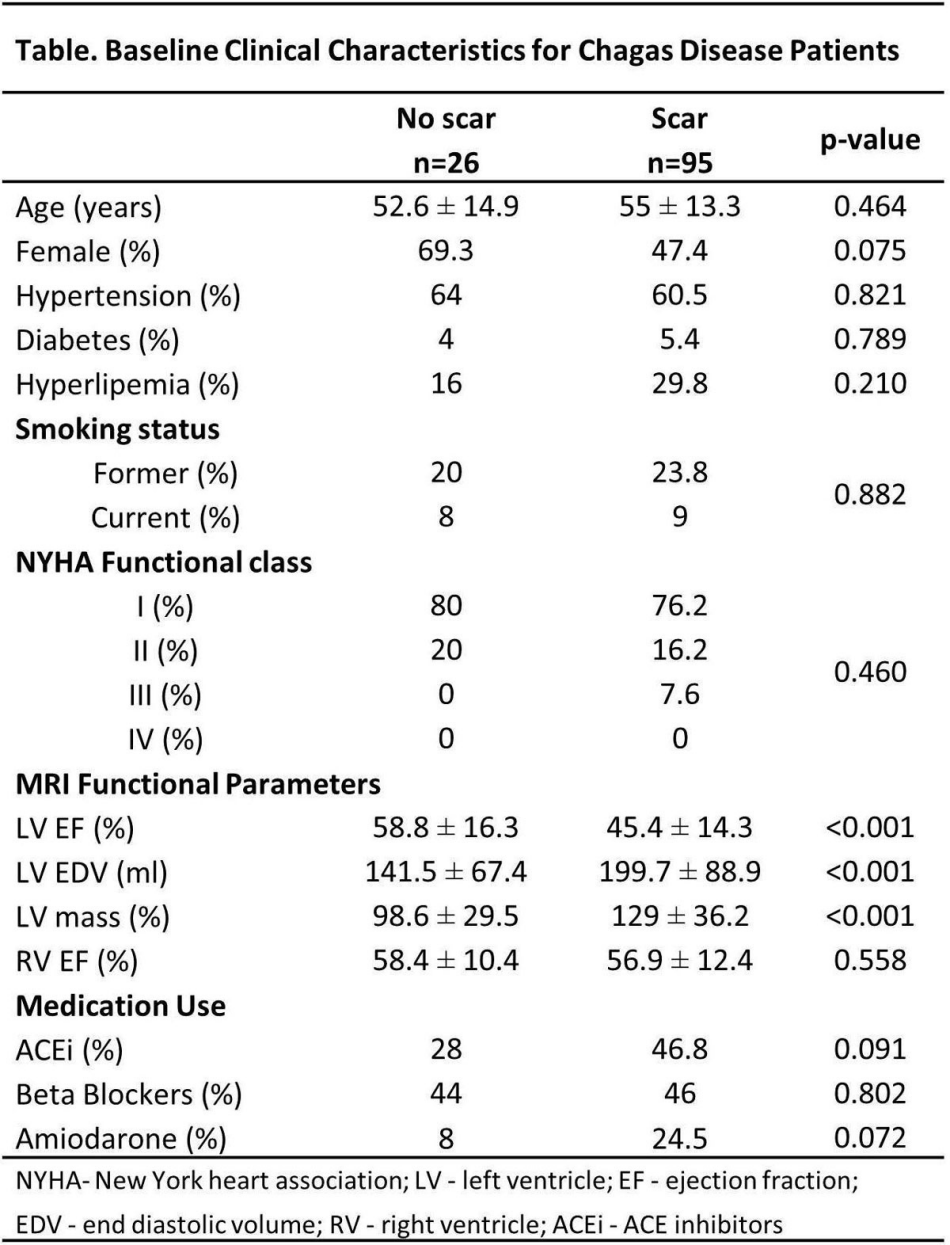
**Baseline clinical characteristics for Chagas Disease patients**.

